# rHGF interacts with rIGF-1 to activate the satellite cells in the striated urethral sphincter in rats: a promising treatment for urinary incontinence?

**DOI:** 10.1007/s00404-018-4930-2

**Published:** 2018-10-10

**Authors:** Xijie Gu, Lailai Fan, Runjiang Ke, Yinghe Chen

**Affiliations:** Department of Urology, The Second Affiliated Hospital and Yuying Children’s Hospital of Wenzhou Medical University, Wenzhou, People’s Republic of China

**Keywords:** Urinary incontinence, Striated urethral sphincter, Satellite cell, Hepatocyte growth factor

## Abstract

**Purpose:**

There are multitudes of factors contributing to urinary incontinence (UI). Dysfunction of the urethral sphincter is one of the common variables. Fortunately, satellite cells, which have the characteristics of stem cells, exist in the striated urethral sphincter. The purpose of the study was to seek whether rHGF combined with rIGF-1 owns the ability to promote the activation, proliferation, and differentiation of satellite cells to potentially improve urinary incontinence.

**Methods:**

The SD rats were randomly divided into four groups and injected with 10 μl rIGF-1, the concentration of which was 50 μg/ml into the urethral wall of the urethral sphincter. Meanwhile, three groups were additionally treated with 10 μl rHGF, the concentration of which was 20, 50, 100 μg/ml. The group injected only with rIGF-1 was used as a control. 30 days later, the urethral tissues were harvested and serially sectioned. Immunofluorescent staining and HE staining were used to detect the activation, proliferation, and differentiation condition of satellite cells. The real-time RT-PCR analysis was applied to explore the potential signaling pathways.

**Result:**

Anti-c-Met antibody-positive cells were discovered in the striated urethral sphincter. Positive expression of c-Met was relatively higher with the treatment of 100 μg/ml rHGF compared to other concentration of rHGF. A similar result was found in additional immunofluorescent staining. The number of newborn myofibers with central nuclei increased as the concentration of rHGF becoming higher. The mRNA expression of ERK1, ERK2 and AKT was comparatively higher with the injection of 50 μg/ml rHGF.

**Conclusion:**

There is supposed to be a synergistic effect between rHGF and rIGF-1 to promote satellite cell to activate, proliferate and differentiate into muscle cells. The urethral sphincter may be induced to renew by the injection of rHGF and rIGF-1 into the urethral wall. It can be used to develop a new therapy for UI.

## Introduction

Urinary incontinence (UI) is defined as any involuntary loss of urine [1]. It is a common health problem and mainly affects all age groups of female populations as well as male populations [2, 3]. According to a population-based cross-sectional study, the prevalence of female UI in Beijing was as high as 38.5%, of which stress urinary incontinence (SUI) was the most common (22.9%) in all subtypes of UI [4]. In a recent review, Buckley et al. reported that the prevalence of any UI ranged from 1 to 39% in men [1]. This disease brings a huge burden on individuals and healthcare systems.

The cause of UI is generally due to the weakness of the urethral sphincter [5]. Nowadays, the therapeutic options for management of UI can be conservative, pharmacological or surgical methods [6]. Patients presenting with UI can choose suitable therapeutic approaches according to their actual condition [6, 7]. Although the current treatments have a certain effect, the most significant condition of urethral sphincter dysfunction has not yet thoroughly been addressed. Urethral sphincter insufficiency still exists commonly [8].

In recent years, stem cell (SC) treatment, including muscle-derived, adipose-derived, bone marrow-derived, even umbilical cord blood-derived, is a promising method to treat UI [9]. SC can differentiate into muscle cells for integrating into urethra intrinsic muscle to remodel urethral sphincter function. However, this therapy requires transplantation of SC via transurethral injection, periurethral injection or intravenous injection [5]. It is said that for periurethral injection, repeated cell injections are required to provide enough stem cells [8]. In addition, another study showed that intravenous injection produced some undesirable side effects in the rat model [10]. The effectiveness and safety of transplanting of SC remain to be tested.

Satellite cells, which are the skeletal muscle stem cells, have been identified to exist in various muscle tissues [11]. When the existing muscle cells are impaired, satellite cells can proliferate and differentiate into myoblasts which then fuse to form new myofibers to renew the muscle function [12]. Therefore, it has the characteristics of SC. Striated urethral sphincter, which is the skeletal muscle portion of the urethral sphincter that surrounds the membranous urethra, plays an important role in urinary control [11]. In recent years, muscle satellite cells are found in the striated urethral sphincter [11, 13, 14]. And it has been identified in rat that the activation of satellite cells can regenerate the urethral sphincter and restore the urinary sphincter function [12, 15].

Exposing the satellite cells from old rats to the serum from young rats can restore the proliferative and regenerative capacity in vitro [16], suggesting that satellite cells require to be exposed to a young systemic environment to activate, proliferate and differentiate [17, 18]. It makes us think about what substances can activate satellite cells. Insulin-like growth factor-1 (IGF-1) is a powerful growth factor of muscle repairment. Several studies reported that satellite cells could be activated by locally and directly injecting IGF-1 [11, 16, 19, 20]. The mechanism might be that IGF-1 activated satellite cells and promoted muscle hypertrophy via the PI3K/AKT pathway or MAPK pathway [17, 21].

Hepatocyte growth factor (HGF), a heparin-binding protein, is crucial in regulating the growth of lots of cells and tissues in vivo. It is reported that the use of HGF did not induce myogenic differentiation of the urethral sphincter satellite cells due to a reduction in phosphorylation of the PI3-kinase pathways [22]. However, another study suggested that both HGF and IGF-1 stimulated the growth of satellite cells [23]. The satellite cell-specific protein, which is called c-Met, is the receptor for HGF [24]. A multitude of researches have identified that IGF-1 can promote the growth of muscle satellite cells. In addition, we have preliminary data demonstrating efficacy of IGF-1 in activating the satellite cells in rat model. Now to verify if HGF and IGF-1 have a synergistic effect to activate the muscle satellite cells and to determine the optimal concentration of HGF for satellite cells growing. In addition, to explore the signal pathways of activating satellite cells after HGF and IGF-1 administration, we conduct the following experiment.

## Materials and methods

### Animal model

12 male SD rats (4 months old) were divided into four groups. After successful isoflurane anesthesia, a midline incision (2–2.5 cm) was made in the low abdomen of the rats, and the prostate was separated to expose the urethra. 10 μl recombinant rat insulin-like growth factor-1 (rIGF-1) and recombinant rat hepatocyte growth factor (rHGF) were injected directly into the urethral wall of the membranous urethra where the urethral sphincter is located using a microinjector. The concentration of rHGF was divided into four subgroups, including 0, 20, 50 and 100 μg/ml. The concentration of rIGF-1 was 50 μg/ml for all the groups. The control group was treated only with the rIGF-1. The incision was closed after injection and the rats were killed and urethral tissues were harvested for a series of studies after 30 days.

### Tissue collection

After the rats were killed with CO_2_, the entire length of the urethra (from the outlet of the bladder to the initiation of the penis) was carefully dissected and removed. Tissues were fixed in 10% formalin, then embedded with paraffin and serially sectioned at 4 μm in the injected region for HE staining and immunofluorescent staining.

### HE staining

Hematoxylin and eosin stains were used for the determination of new myofibers. Observation under a microscope of 200 and 400 times: myofibers containing central nuclei were assumed to be new myofibers. One slice was taken from each rat; each section observed three fields randomly. The number of newborn myofibers was counted from each field, and the effect of different rHGF concentrations on newborn myofibers growing was analyzed.

### Immunohistochemistry

To detect c-Met, sections were incubated in block (5% BSA) for 45 min at 37 °C. The sections were then incubated with 1:200 dilution c-Met antibody (abcam; ab51067) at 4 °C overnight, followed by three PBS washes and incubated with Alexa Fluor™ 488 goat anti-rabbit IgG, 1:1000 dilution, (Invitrogen) for 1 h at 37 °C. To detect MYH3 and NCAM, the procedure was similar, just replaced the c-Met antibody with the MYH3 antibody (Santa Cruz; sc-53091) and NCAM antibody (abcam; ab9018). DAPI was used to the sections for 5 min to identify nuclei. To detect Ki67, after cultivated with BSA for 45 min, the sections were incubated overnight at 4 °C with the Ki67 antibody (CST; 12075S), Propidium bromide (PI) was applied to the sections for 30 min to identify nuclei. Fluorescent microscopy was used to identify the efficacy of rHGF on satellite cell growth. Cells were stained green with c-Met primary antibody and Alexa Fluor^TM^ 488 secondary antibodies. Nuclei were stained to appear blue with DAPI. The co-localization of c-Met staining and DAPI demonstrated satellite cells. Likewise, The co-localization of MYH3 staining and DAPI, NCAM staining and DAPI, Ki67 staining and PI demonstrated satellite cells, respectively. Images were captured with Nikon ECLIPSE Ti and Lecia TCS SP8 Camera system. Image J was used to analyze the co-localization of c-Met, MYH3, NCAM and Ki67 positive staining with nuclei. Image-pro-plus was applied to count the mean density of immunofluorescent staining.

### Real-time RT-PCR analysis

Total RNA was extracted from tissues with total RNA isolation kit (Generay) according to the manufacturer’s instructions. Then, RNA was converted into complementary DNA (cDNA) with the RevertAidFirst strand cDNA synthesis kit (Thermo) according to the manufacturer’s instructions. Real-time RT-PCR was performed to amplify the cDNA with the SYBR Green SuperMix (BIO-RAD) method. The relative quantification of ERK1, ERK2, AKT, MyoD, MyoG and MRF4 mRNA expression was calculated automatically using a CFX connect real-time PCR system with the 2$^{{ - \Delta \Delta C_{\text{t}} }}$ method. The PCR thermocycler parameters were 95 °C for 15 s, 59 °C for 30 s and 72 °C for 30 s, for 40 cycles. Melt curve 65–95 °C: increment 0.5 °C for 5 s. The primers used in this article are listed below: ERK1, 5′-CGGATTGCTGACCCTGAGCA-3′ (forward) and 5′-CAATGGATTTGGTGTAGCCCTTG-3′ (reverse); ERK2, 5′-CAAGCCTTCCAACCTCCTGC-3′ (forward) and 5′-GGATGCAGCCCACAGACCAAAT-3′ (reverse); AKT, 5′-TTTATTGGCTACAAGGAACGGC-3′ (forward) and 5′-CAGGCAGCGGATGATGAAGGTG-3′ (reverse); MyoD, 5′-CCTGGGCGTGTAAGGTGT-3′ (forward) and 5′-GTAGGCGCTCAATGTACTGGAT-3′ (reverse); MyoG, 5′-GGCAGCCACCATGCGTGAG-3′ (forward) and 5′-GGGTAGCCGCTGGTTCG-3′ (reverse); MRF4, 5′-TGAAGCGTAGAACTGTGGCC-3′ (forward) and 5′-GGGTTTGTAGCTGTAAGGGT-3′ (reverse).

### Statistical analysis

One-way analysis of variance was performed to determine the significant difference between four groups. Data were presented as means ± SE.

## Results

### c-Met antibody staining

**Fig. 1:**
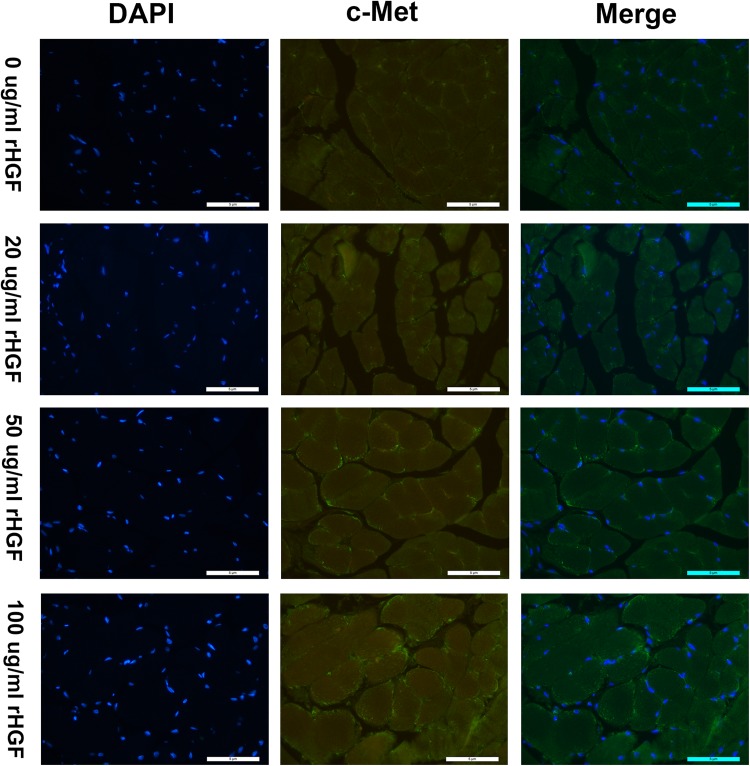
Green glowing dots represented positive staining with c-Met antibody in the urethral sphincter tissue, demonstrating the presence of satellite cells. Nuclei were stained blue with DAPI. The merged images showed co-localization

**Fig. 2:**
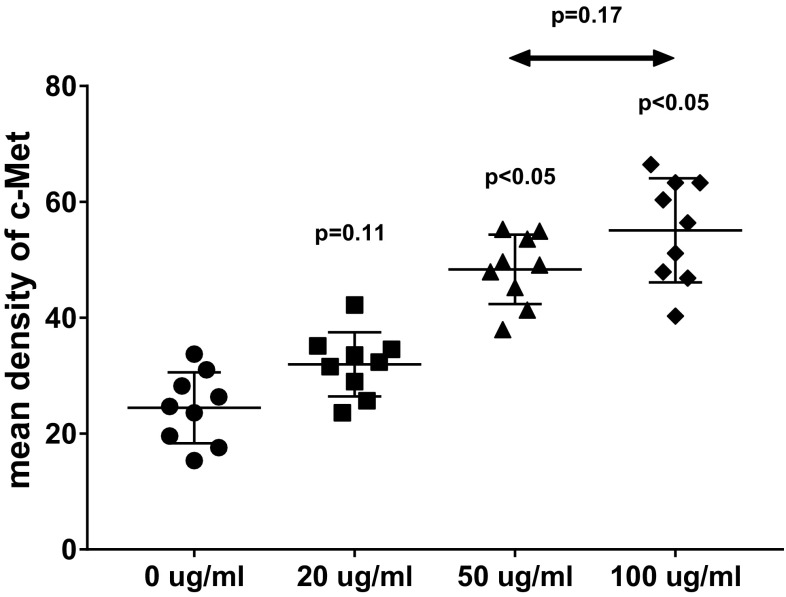
Determination of whether rHGF plays a role in promoting c-Met expression. The mean fluorescence density in different concentration groups was detected. 20, 50, 100 μg/ml rHGF groups are compared with 0 μg/ml rHGF group. Additional contrast is performed between 50 and 100 μg/ml rHGF groups. *p* < 0.05 represents significant difference

It was previously demonstrated that satellite cells exist in the urethral sphincter [11]. The c-Met protein played an important role in the proliferation of muscle cells; so, the expression level of c-Met was relatively high in newborn muscle tissue. The c-Met was also a receptor tyrosine kinase activated by rHGF and a marker of satellite cells throughout its activation and proliferation [25]. To detect the effect of rHGF combined with rIGF-1 on satellite cells growing, the urethral muscular tissue was stained with an antibody to c-Met. As shown in Fig. 1, green glowing dots represented the expression of c-Met. All nuclei were stained blue with DAPI. Satellite cells were, thus, double stained. In the merge photograph, as the concentration of rHGF increased from 0 to 100 μg/ml, the positive expression of c-Met was added. To test whether the positive expression between different concentration groups had a significant difference. The mean density of c-Met immunofluorescent staining was counted in three random fields from each section. Therefore, nine records for each group were collected and the average of mean density was calculated (Fig. 2). Thirty days after treatment with 100 μg/ml rHGF, the average of the mean density of positive expression of c-Met increased by 125.8% (55.1 ± 9.0 vs 24.4 ± 6.1, *p* < 0.05), comparing to the control group; while the 50 μg/ml rHGF group increased by 98.0% (48.3 ± 6.0 vs 24.4 ± 6.1, *p* < 0.05) in comparison with the control group. However, the 100 μg/ml rHGF group showed no significant change from 50 μg/ml rHGF group (55.1 ± 9.0 vs 48.3 ± 6.0, *p* = 0.17). Meanwhile, there was no significant difference between the 20 μg/ml rHGF group and the control group (31.9 ± 5.5 vs 24.4 ± 6.1, *p* = 0.11).

### Ki-67, MYH3, and NCAM antibody staining

**Fig. 3:**
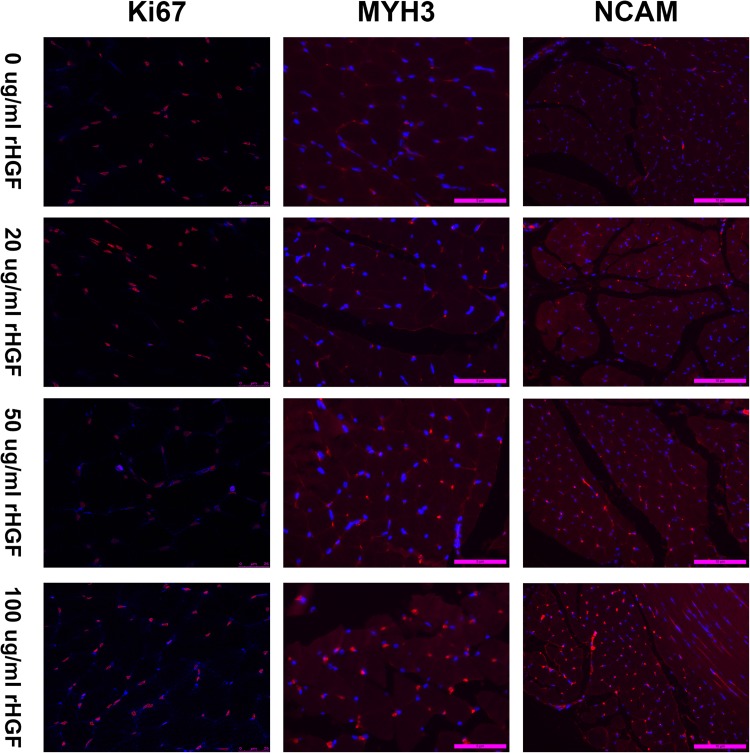
In Ki-67 staining, all nuclei were stained red with PI. The satellite cells expressing Ki67 were stained blue with Ki67 antibody. In MYH3 and NCAM staining, all nuclei were stained blue with DAPI. The satellite cells were stained red with MYH3 and NCAM antibody. All of the images are merged and show co-localization

To further verify if rHGF combined with rIGF-1 really contributed to the activation, proliferation, even differentiation of satellite cells, other immunofluorescence staining experiments were applied. Ki-67 is strictly associated with cell proliferation and represents the proliferation condition of satellite cells. It is a proliferation marker [26, 27]. MYH3 (myosin heavy chain 3) shows the proliferation and differentiation status of satellite cell after its activation and is routinely used to reflect the condition of skeletal muscle repair and renewal [28, 29]. NCAM is a recognized marker of satellite cells and indicates the activation and proliferation of satellite cell in the urethral sphincter [30–32]. These three indicators will further demonstrate the relationship between the amount of rHGF administering and the condition of muscle satellite cells activation, proliferation, and differentiation. In Ki-67 antibody staining, the sections were stained with an antibody to Ki-67. Nuclei were stained with PI. In MYH3 and NCAM antibody staining, the sections were stained with an antibody to MYH3 and NCAM. DAPI was used to identify all nuclei. The result showed that with the concentration of rHGF increasing, the positive expression of Ki67, MYH3, and NCAM became stronger (Fig. 3). The result of the three experiments was coincident with c-Met staining. It may indicate that rHGF combined with rIGF-1 will contribute to the activation, proliferation, and differentiation of satellite cells. Besides, the rHGF may have a dose-dependent effect on satellite cells.

### HE staining

**Fig. 4:**
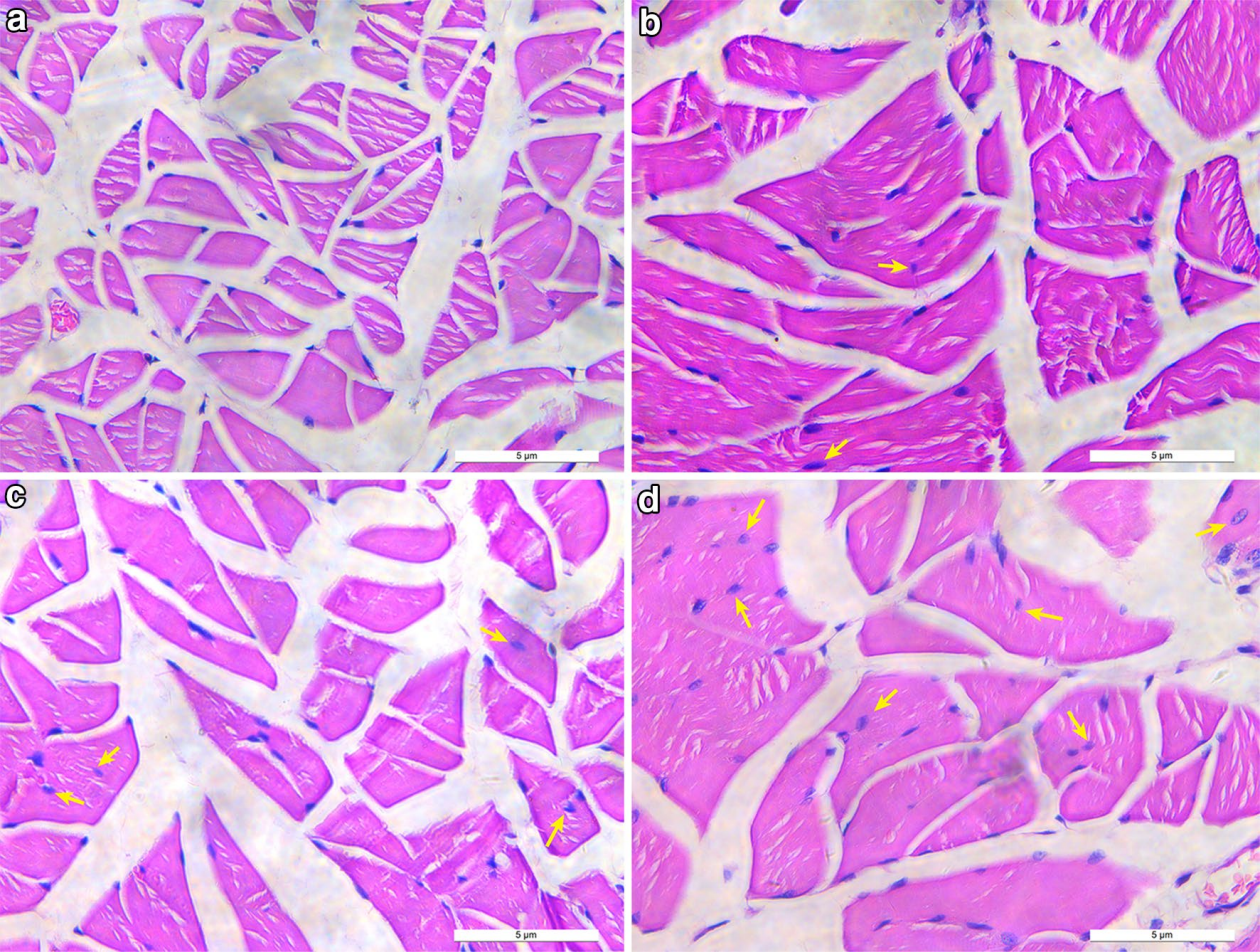
The images show the number of new myofibers in the urethral sphincter with the treatment of 0, 20, 50, 100 μg/ml rHGF (**a**–**d**) combined with 50 μg/ml rIGF-1 under 400 times microscope observation. The new skeletal muscle cells with central nuclei (arrows) are supposed to be differentiated from satellite cells

**Fig. 5:**
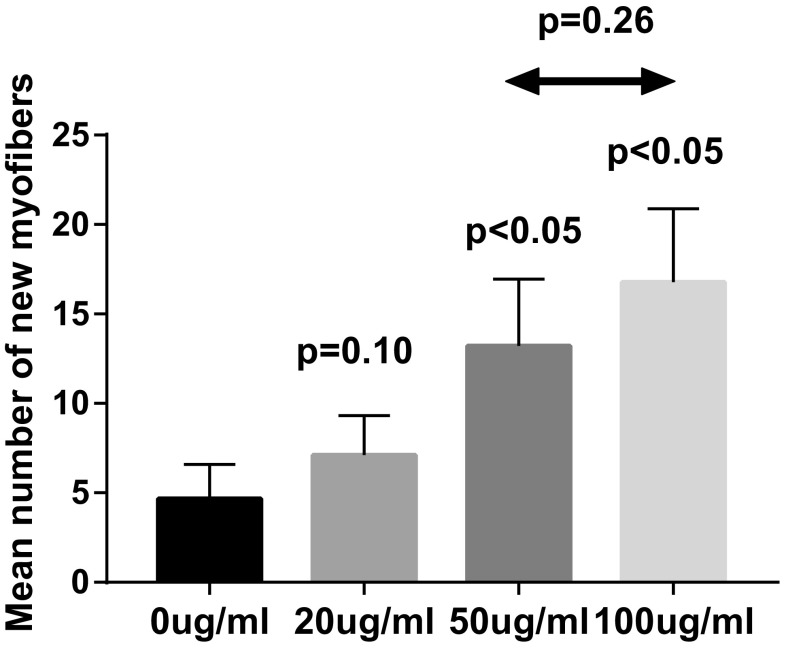
To determine whether rHGF has an effect on inducing satellite cells to muscle cells, the number of newborn skeletal muscle cells in different concentration groups were observed under 200 times microscope. *p* < 0.05 represents significant difference

To certify if the satellite cells differentiate into new myofibers after injection of the rHGF and rIGF-1 and to exhibit the effects of different concentration of rHGF on promoting satellite cells differentiation in the urethral sphincter, HE staining was applied to the sections originated from the urethral tissues. As shown in Fig. 4, under 400 times microscope observation, cells with centrally located nuclei represented regenerated muscle cells [33]. The result demonstrated that the urethral sphincter generated more newborn muscle cells with the concentration of rHGF becoming higher. In addition, three random fields were taken from each slice under 200 times microscope observation and the number of new myofibers was calculated in the four groups. The result was shown by the way of the mean value of newborn myofibers (Fig. 5). The number of new myofibers increased by 257.4% (16.8 ± 4.1 vs 4.7 ± 1.9, *p* < 0.05) in 100 μg/ml rHGF group in comparison with the control group. However, there was no significant difference between 100 μg/ml rHGF group and 50 μg/ml rHGF group (16.8 ± 4.1 vs 13.2 ± 3.7, *p* = 0.26).

### Real-time RT-PCR analysis

**Fig. 6:**
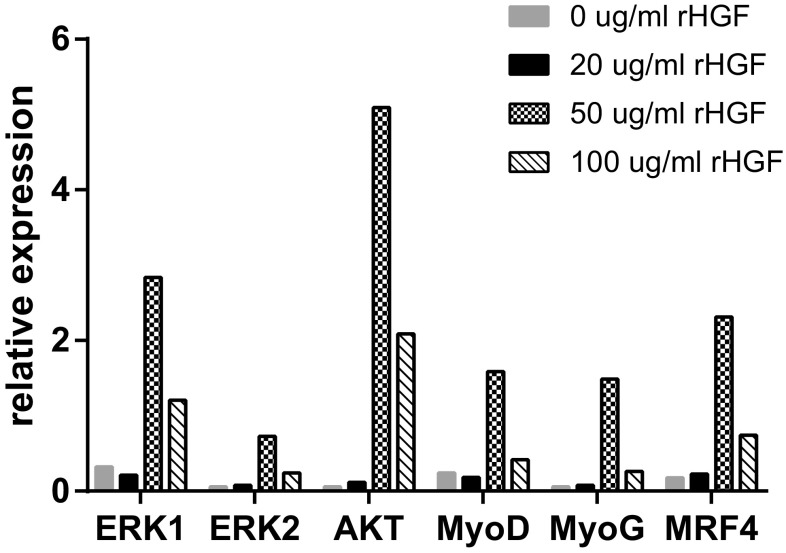
Identification of the mechanism of satellite cell activation, proliferation, and differentiation. RT-PCR was used to detect the expression of genes such as ERK1, ERK2, AKT, MyoD, MyoG, and MRF4. The relative expression of these genes is shown

Previous studies have demonstrated that rIGF-1 possessed a critical status in activating the PI3K/Akt signaling pathways as well as the MAPK signaling pathways to facilitate the proliferation and differentiation of satellite cells [23, 34, 35]. We hypothesized that rHGF had a synergistic effect with rIGF-1. However, some articles denoted that different concentrations of rHGF had different effects on satellite cells growing and the induction function was mainly associated with ERK1, ERK2, and AKT [36]. In this paper, we compared the expression of each gene under different concentrations of rHGF in the premise of controlling the concentration of rIGF-1 all the same. To explore the signal pathways of satellite cell activation, proliferation, and differentiation after treatment with rHGF and rIGF-1. Real-time RT-PCR was used to detect the mRNA levels of ERK1, ERK2, AKT, MyoD, MyoG, and MRF4. The relationship between the rHGF concentration and the mRNA expression levels of above genes will also be assessed guiding to find out the optimal concentration of rHGF for satellite cell proliferation and differentiation. As shown in Fig. 6, the mRNA expression of all detective genes was relatively higher in 50 μg/ml rIGF1 + 50 μg/ml rHGF group than in other concentration groups. The expression of Akt in 50 μg/ml rHGF group was more than twofold higher than in 100 μg/ml rHGF group. Nevertheless, the expression of genes in the 100 μg/ml rHGF groups was still higher compared with the 0 and 20 μg/ml rHGF groups. Thus, it was speculated that rHGF may promote satellite cells proliferation and division into new mature myocytes through MAPK (ERK1, ERK2) and PI3K/Akt pathways. MyoD, MyoG and MRF4, the myogenic regulatory genes expressing abundantly in muscle development, may play an important role in the transcriptional regulation of activating the resting muscle satellite cells. According to the result, it seemed that 50 μg/ml rHGF was probably the most appropriate concentration to promote muscle satellite cell growing compared to other concentration.

## Discussion

The urethral sphincter locates in the mid-urethra and includes outer layer of striated muscle and inner layer of smooth muscle. The smooth muscle layer divides into the internal longitudinal smooth muscle layer and the external circular smooth muscle layer. The striated muscle of urethral sphincter surrounds the two smooth muscle layers, contributing to urethral closure to stop urinating from the bladder. The damage to the striated urethral sphincter during surgery, pelvic trauma and vaginal delivery, as well as muscle degeneration and pelvic floor relaxation when individuals getting older, can result in UI. The striated urethral sphincter deterioration is strongly associated with UI. However, previous studies have demonstrated that urethral sphincter do contain satellite cells, and satellite cells are mainly enriched in the striated urethral sphincter [11]. Satellite cells are muscle-derived stem cells. When they are appropriately stimulated, they can be activated, and then proliferate and differentiate into mature muscle cells. In the present study, the new myofibers, characterized by central nuclei, were discovered in urethral tissues after treatment of rHGF and rIGF-1. It further validated this notion. Based on this principle, inducing satellite cells to differentiate into myofibers to strengthen the urethral sphincter has great potential to improve UI symptom.

It has been generally assumed that rIGF-1 can activate satellite cells. Some articles reported the essential role of rHGF on satellite cells [37]. However, few explored the molecular mechanism of satellite cells growth and the most appropriate concentration of rHGF on satellite cells. In this study, we selected male rats as experimental subjects. We did not choose female rats because it was more difficult to pinpoint the urethral sphincter of female rats. Nevertheless, both female and male rats contained urethral sphincter. Injecting the rHGF and rIGF-1 directly into the urethral sphincter of the rats, we compared the effects of different concentrations of rHGF on satellite cell proliferation and differentiation. In the immunofluorescent staining, we found that as the dose of rHGF increases, the fluorescence intensity also increases. It was implied that rHGF combined with rIGF-1 could be effective in activating satellite cells and promoting its proliferation and differentiation. In addition, rHGF seemed to have a dose-dependent effect on satellite cells. However, from the results of the mean fluorescence density, although the average of the mean density in 100 μg/ml rHGF group was higher than 50 μg/ml rHGF group, there was no significant difference between the two groups. The result of HE staining showed that satellite cells do differentiate into myocytes and further prove the contributing role of rHGF. Similarly, no difference was found in the number of new muscle cells between 50 and 100 μg/ml rHGF groups. Thus, we considered that 50–100 μg/ml rHGF was the relative appropriate concentration to stimulate satellite cells. In the real-time RT-PCR analysis, we detected the expression of different genes such as ERK1, ERK2, AKT, MyoD, MyoG and MRF4 after rHGF and rIGF-1 treatment. However, we found that the 50 μg/ml group, not the 100 μg/ml group, had a higher expression level of each gene. We hypothesized that this was because the large differences in animal tissue samples, which led to intra-group biases, or satellite cells achieved the proliferation and differentiation procedure through other molecular signal pathways without the genes above. It also indirectly proved that the most suitable concentration of rHGF was between 50 and 100 μg/ml. For rats, the clear optimum concentration of rHGF for satellite cell growth requires more experiments to discover.

The rats used in our experiment were 4-month-old younger rats. The result part of c-Met staining showed that the positive expression in the 100 μg/ml rHGF group was increased by 125.8% compared with the 0 μg/ml rHGF group. What is more, the HE staining counting newborn cells found that 100 μg/ml rHGF group increased by 257.4% in contrast with 0 μg/ml rHGF group. Here, we have to admit that there may be a certain amount of error due to the small number of rats and the less materials harvested. But the results still may prove that rHGF interacts with rIGF-1 to activate the satellite cells and promote the generation of new muscle cells in the striated urethral sphincter. In humans, UI is usually a disease that occurs in adult or the old ones. We next consider using aged rats and increasing the number of samples to thoroughly study the role of rHGF on satellite cells.

Since this study was an animal experiment with a small sample capacity, more functional studies still would be needed to be carried out to validate the contractility and physiological function of the urethral sphincter after rHGF and rIGF-1 administration. Because of the small number of experimental rats, only few tissues were acquired; so, we gave up the western blotting and only used RT-qPCR analysis to elucidate genes expression. More experiments were obviously needed to investigate the particular mechanism of satellite cells activation, proliferation, and differentiation procedure. Otherwise, our study was just an animal experiment. The safety and efficacy of HGF and IGF-1 treatment on human urethral sphincter should be tested necessarily by more experiments.

## Conclusion

Satellite cells do exist in the urethral sphincter. rHGF may have a stimulating effect on satellite cells and interacts with rIGF-1 to promote satellite cells differentiating into new muscle cells. The treatment of rIGF-1 and rHGF may partly renew the striated sphincter. These findings contribute to our understanding of rHGF combined with rIGF-1 to the urethral sphincter regeneration in this animal model. It may be possible to consider the injection of rHGF and rIGF-1 into the urethral sphincter as a promising therapeutic approach to relieve symptoms of urinary incontinence, the root cause of which is the dysfunction of urethral sphincter.
